# Supplementary material for: Pan-urologic cancer genomic subtypes that transcend tissue of origin
Source: Nat Commun. 2017 Aug 4;8:199. doi: 10.1038/s41467-017-00289-x (PMC5543131; doi:10.1038/s41467-017-00289-x)
Supplement: Supplementary file 1 — Supplementary Information [file 41467_2017_289_MOESM1_ESM.pdf]

File name: Supplementary Information
Description: Supplementary Figures and Supplementary References

File name: Supplementary Data 1
Description: Related to Figure 2. TCGA urologic cancer sample annotation table, by patient. Provided as an Excel file. Patient-level table includes the following types of information: clinical information, any notes made from previous TCGA marker studies, molecular profiling data platforms available for the tumor sample, molecular status or measurement regarding selected gene features highlighted in the present study, and membership in various molecular profile-based classification schemes (by individual platform or multiplatform analyses).

File name: Supplementary Data 2
Description: Related to Figure 2. mRNA features that help to distinguish between the pan-urologic cancer genomic subtypes. Provided as an Excel file. Includes a table of the differential expression patterns for the top 900 genes (from Figure 2b) that help to distinguish between the nine COCA-based genomic subtypes (for each subtype, showing the top 100 genes most differentially in the given subtype versus the rest of the tumors).

File name: Supplementary Data 3
Description: Related to Figure 2. DNA methylation features that help to distinguish between the pan-urologic cancer genomic subtypes. Provided as an Excel file. Includes DNA methylation (beta) values for the top 2000 genomic loci with the highest variability in DNA methylation patterns (for combined 27K/450K dataset, with values then centered within each cancer type) across tumors (from Figure 2c).

File name: Supplementary Data 4
Description: Related to Figure 2. For top DNA methylation features, correlations with mRNA expression across tumors. Provided as an Excel file. For the top 2000 genomic loci with the highest variability in DNA methylation patterns (Supplementary Data 3), DNA methylation (beta) values are correlated with mRNA expression of the associated gene by Pearson's. Correlations are based on the sets of values that were first centered within each cancer type. The features with significant anti-correlations between mRNA and DNA methylation are listed in a separate table.

File Name: Supplementary Data 5
Description: Related to Figure 4. Somatic mutations events for genes of interest. Provided as an Excel file. Genes represented are those featured in Figure 4 and Supplementary Figure 4.

File Name: Peer Review File
Description:

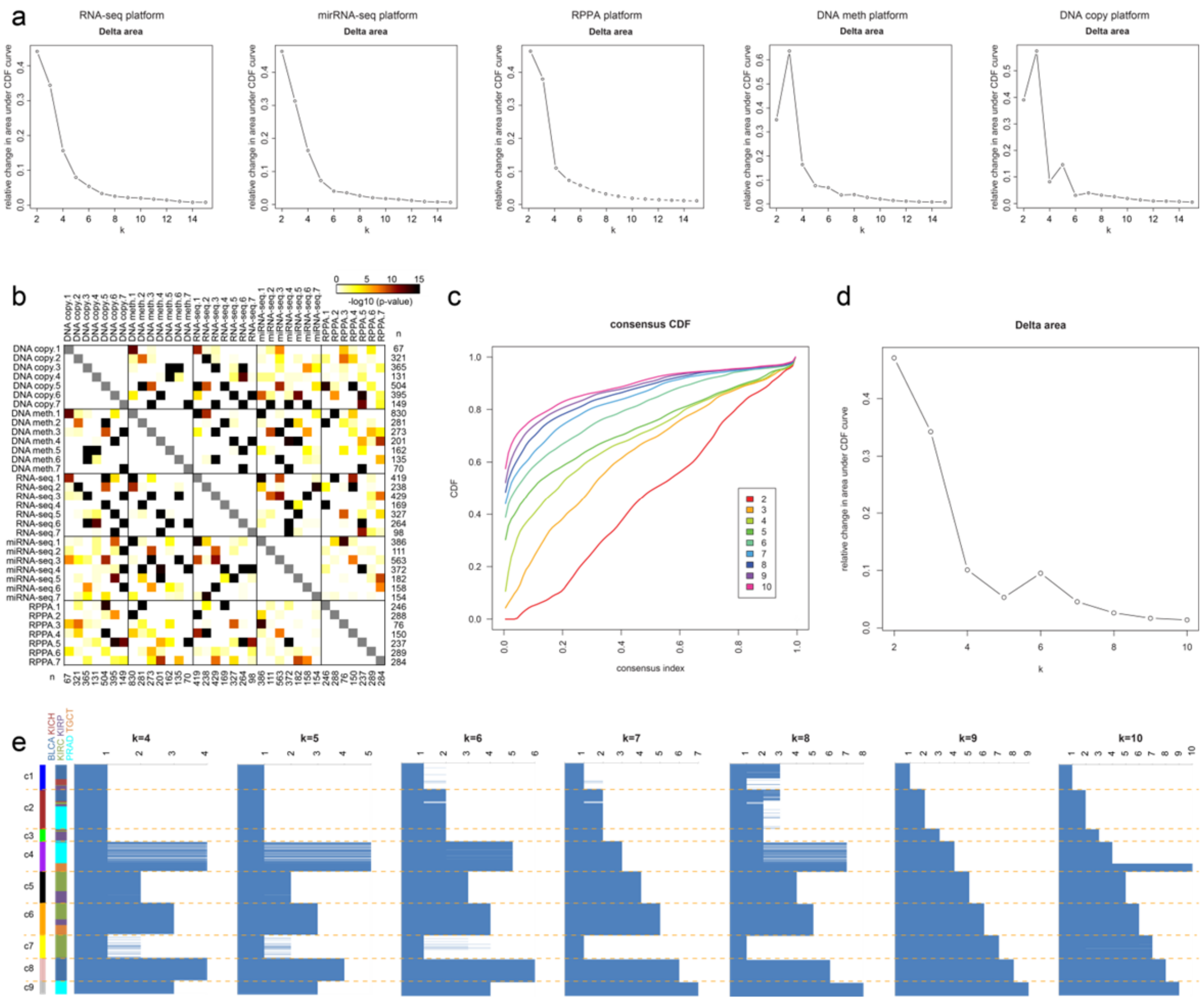

Supplementary Figure 1, related to Figure 1. Derivation of genomic subtypes of urologic cancer cases in TCGA cohort, on the basis of information from multiple data platforms. (a) For each data platform (DNA copy, DNA methylation, miRNA expression, mRNA expression, RPPA), subtypes from k=2 to k=15 were defined using ConsensusClusteringPlus (see Methods). For each respective platform, delta area plot graphics show the relative change in area under the CDF curve comparing k and k − 1. For k = 2, there is no k -1, so the total area under the curve rather than the relative increase is plotted. This graphic allows one to determine the relative increase in consensus and determine k at which there is no appreciable increase. **(b)** Significance of overlap (by one-sided Fisher's exact test) between the subtype assignments made between the various data platforms (DNA copy, DNA methylation, miRNA expression, mRNA expression, RPPA). To define multiplatform-based molecular subtypes, each individual platform was first used to define seven different subtypes of urologic cancer spanning BLCA, KICH, KIRC, KIRP, PRAD, and TGCT projects (for expression and methylation datasets, values were first centered within cancer type as described in

Methods). **(c)** To provide an integrated level of assessment of these platform-specific subtypes, subtype calls made by the different molecular platforms were combined by a "cluster of clusters analysis" (COCA) approach (see also main Figure 2a). Graphic shows the cumulative distribution functions (CDFs) of the consensus matrix for each k (indicated by colors) in the COCA consensus clustering results, estimated by a histogram of 100 bins. This graphic was used as a guide to determine at what number of clusters, k, the CDF reaches an approximate maximum. **(d)** Delta area plot graphic showing the relative change in area under the CDF curve comparing k and k − 1. For k = 2, there is no k -1, so the total area under the curve rather than the relative increase is plotted. This graphic allows one to determine the relative increase in consensus and determine k at which there is no appreciable increase. **(e)** For the set of 1954 urologic cancer cases (ordered by genomic subtype according to the k=9 solution), subtype assignments as made on the basis of differing COCA k solutions (from k=4 to k=10) are shown. At k=9, BLCA cases are subdivided between three subtypes, and a fourth KIRP subtype (c3) is defined. At k=10, the k=9 c4 TGCT cases form their own subtype distinct from that of the other c4 cases.

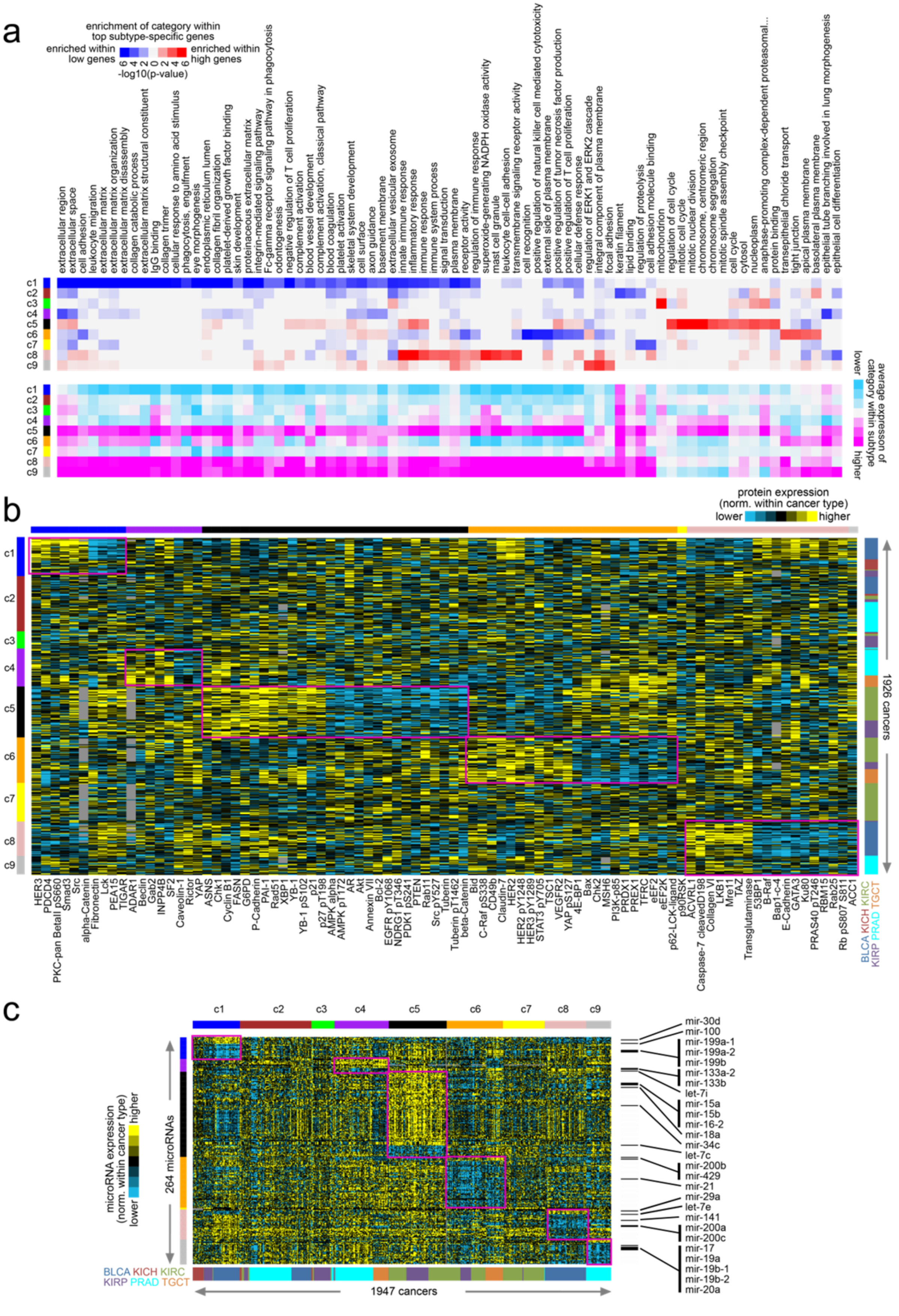
a
enrichment of category within
top subtype-specific genes
enriched within
low genes
enriched within
high genes
6 4 2 0 2 4 6
-log10(p-value)
extracellular region
extracellular space
cell adhesion
leukocyte migration
extracellular matrix
extracellular matrix organization
extracellular matrix disassembly
collagen catabolic process
extracellular matrix structural constituent
IgG binding
collagen trimer
cellular response to amino acid stimulus
phagocytosis, engulfment
eye morphogenesis
endoplasmic reticulum lumen
collagen fibril organization
platelet-derived growth factor binding
skin development
proteinaceous extracellular matrix
integrin-mediated signaling pathway
Fc-gamma receptor signaling pathway in phagocytosis
odontogenesis
negative regulation of T cell proliferation
complement activation
blood vessel development
complement activation, classical pathway
blood coagulation
platelet activation
skeletal system development
cell surface
axon guidance
basement membrane
extracellular vesicular exosome
innate immune response
inflammatory response
immune response
immune system process
signal transduction
plasma membrane
receptor activity
regulation of immune response
superoxide-generating NADPH oxidase activity
mast cell granule
leukocyte cell-cell adhesion
transmembrane signaling receptor activity
cell recognition
positive regulation of natural killer cell mediated cytotoxicity
external side of plasma membrane
positive regulation of tumor necrosis factor production
positive regulation of T cell proliferation
cellular defense response
regulation of ERK1 and ERK2 cascade
integral component of plasma membrane
focal adhesion
keratin filament
lipid binding
regulation of proteolysis
cell adhesion molecule binding
mitochondrion
regulation of cell cycle
mitotic cell cycle
mitotic nuclear division
chromosome, centromeric region
chromosome segregation
mitotic spindle assembly checkpoint
cell cycle
cytosol
nucleoplasm
anaphase-promoting complex-dependent proteasomal...
protein binding
transepithelial chloride transport
tight junction
apical plasma membrane
basolateral plasma membrane
epithelial tube branching involved in lung morphogenesis
epithelial cell differentiation
c1
c2
c3
c4
c5
c6
c7
c8
c9
average expresson of
category within subtype
lower
higher
b
protein expression
(norm. within cancer type)
lower
higher
c1
c2
c3
c4
c5
c6
c7
c8
c9
1926 cancers
HER3
PDCD4
PKC-pan BetaII pS660
Smad3
Src
alpha-Catenin
Fibronectin
Lck
PEA15
TIGAR
ADAR1
Beclin
Gab2
INPP4B
SF2
Caveolin-1
Rictor
YAP
ASNS
Chk1
Cyclin B1
FASN
G6PD
P-Cadherin
PAI-1
Rad51
XBP1
YB-1
YB-1 pS102
p21
p27 pT198
AMPK alpha
AMPK pT172
AR
Akt
Annexin VII
Bcl-2
EGFR pY1068
NDRG1 pT346
PDK1 pS241
PTEN
Rab11
Src pY527
Tuberin
Tuberin pT1462
beta-Catenin
Bid
C-Raf pS338
CD49b
Claudin-7
HER2
HER2 pY1248
HER3 pY1289
STAT3 pY705
TSC1
VEGFR2
YAP pS127
4E-BP1
Bax
Chk2
MSH6
PI3K-p85
PRDX1
PREX1
TFRC
eEF2
eEF2K
p62-LCK-ligand
p90RSK
ACVRL1
Caspase-7 cleavedD198
Collagen VI
LKB1
Mre11
TAZ
Transglutaminase
53BP1
B-Raf
Bap1-c-4
E-Cadherin
GATA3
Ku80
PRAS40 pT246
RBM15
Rab25
Rb pS807 S811
ACC1
BLCA KICH KIRC
KIRP PRAD TGCT
c
c1
c2
c3
c4
c5
c6
c7
c8
c9
microRNA expression
(norm. within cancer type)
lower
higher
264 microRNAs
mir-30d
mir-100
mir-199a-1
mir-199a-2
mir-199b
mir-133a-2
mir-133b
let-7i
mir-15a
mir-15b
mir-16-2
mir-18a
mir-34c
let-7c
mir-200b
mir-429
mir-21
mir-29a
let-7e
mir-141
mir-200a
mir-200c
mir-17
mir-19a
mir-19b-1
mir-19b-2
mir-20a
BLCA KICH KIRC
KIRP PRAD TGCT
1947 cancers

Supplementary Figure 2, related to Figure 2. Additional features characterizing the multiplatform-based genomic subtypes of pan-urologic cancer in TCGA cohort. (a) Enriched gene categories associated with genomic subtypes. For the top differentially expressed genes associated with each subtype (the genes represented in main Figure 2b), represented categories by Gene Ontology (GO) were assessed, with "high" genes evaluated separately from "low" genes. P-values by one-sided Fisher's exact test. GO terms with significance level of $p<0.0005$ (involving at least three genes) for any one gene set are represented here. Top panel shows $-\log_{10}$(p-value) by GO term and subtype (red, associated with the subtype-specific "high" genes; blue, associated with the subtype-specific "low" genes). Bottom panel shows the average relative expression within a given subtype, for all genes falling under a given GO term category. **(b)** Differential protein expression patterns (by RPPA platform, values normalized within each main cancer type), representing a set of genes that help to distinguish between the nine subtypes (for each subtype, showing the top significant correlates found for the given subtype versus the rest of the tumors, where the significant of Pearson's correlation exceeded p<1E-6). **(c)** Differential microRNA expression patterns (values normalized within each main cancer type), representing a set of genes that help to distinguish between the nine subtypes (for each subtype, showing the top significant correlates found for the given subtype versus the rest of the tumors, where the significant of Pearson's correlation exceeded p<1E-9).

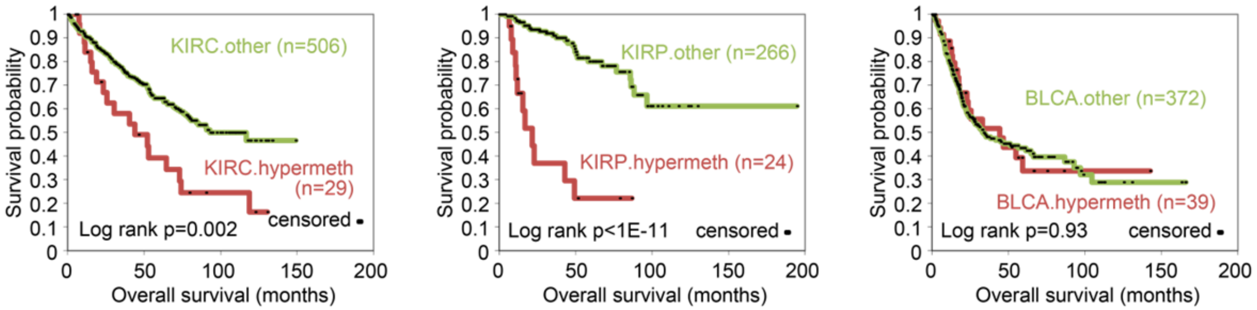

Supplementary Figure 3, related to Figure 3. Differences in patient overall survival, according to the presence or absence of high overall DNA methylation patterns. Within TCGA KIRC cases (left panel), KIRP cases (middle panel), and BLCA cases (right panel), respective differences in patient overall survival, comparing cases with high overall DNA methylation patterns (defined here as >35% of methylation probes in the tumor profile having beta value>0.3) to the rest of the cases. P-values by Log rank test.

% cases by project/subtype

Pathway	Gene	% cases	BLCA.c1	BLCA.c2	BLCA.c8	KICH.c1	KIRC.c5	KIRC.c6	KIRC.c7	KIRP.c1	KIRP.c3	KIRP.c5	KIRP.c6	PRAD.c2	PRAD.c4	PRAD.c9	TGCT.c4	TGCT.c6
Chromatin Modifiers	*KMT2D*	9.3	29.2	33.7	28.3	4	2	1.8	2.4	10.3	4.1	7.1	4.3	5.1	7.3	2.6	1.4	2.5
	KMT2C	7.5	24.8	16.9	19.3	2	2	5.5	3.6	2.6	6.8	9.1	4.3	6.2	5.6	1.7	0	2.5
	KDM6A	6.8	34.5	30.3	19.8	0	0	0	0.6	2.6	11	3	0	1.5	4.5	0	0	0
	SETD2	6.3	3.5	6.7	8.6	2	19.3	4.5	16.4	2.6	1.4	15.2	0	1.5	1.7	1.7	0	0
	EP300	4.7	12.4	16.9	18.2	0	3.3	0.9	0.6	7.7	5.5	3	2.1	0.5	1.7	1.7	0	1.3
	CREBBP	3.6	14.2	7.9	14.4	0	0.7	0.9	0	0	1.4	3	2.1	1	1.1	0.9	1.4	1.3
	KMT2A	3.2	6.2	11.2	15	0	2	0	1.2	0	0	3	4.3	0.5	2.8	0	0	0
	SRCAP	3.2	8	6.7	10.2	2	0.7	1.8	1.2	0	2.7	4	2.1	0.5	2.8	0.9	1.4	5
	KDM5C	2.2	1.8	1.1	0.5	2	9.3	3.6	8.5	7.7	0	0	2.1	0	0	0	0	0
	NSD1	1.9	5.3	6.7	5.3	0	0.7	0.9	0.6	2.6	2.7	1	0	0.5	1.1	0	0	0
	KMT2B	1.9	2.7	6.7	6.4	2	0.7	0	1.2	7.7	2.7	1	0	0.5	1.1	0	2.9	0
	KMT2E	1.9	7.1	3.4	6.4	0	1.3	0.9	0	2.6	1.4	1	4.3	0.5	1.7	0.9	0	0
	KDM5A	1.8	4.4	6.7	6.4	2	1.3	0	0.6	0	0	1	4.3	0.5	1.1	0	0	1.3
	KDM5B	1.3	2.7	3.4	4.3	0	0.7	0.9	0.6	2.6	1.4	0	2.1	0	1.1	0	0	1.3
	EHMT2	1.1	2.7	4.5	2.7	0	0.7	0.9	0	2.6	1.4	2	0	0	1.1	0.9	0	0
SWI/SNF Complex	*PBRM1*	11.3	2.7	4.5	5.9	2	36	31.8	50.9	2.6	0	9.1	2.1	0	0.6	0.9	0	1.3
	ARID1A	7.3	25.7	30.3	23.5	0	3.3	2.7	4.2	2.6	4.1	8.1	0	0	1.7	0.9	0	1.3
	ARID2	2.7	8	4.5	10.2	0	0	1.8	0.6	5.1	0	4	0	0.5	2.8	0.9	0	0
	SMARCA4	2.6	5.3	4.5	7	0	1.3	2.7	3	5.1	8.2	2	4.3	0	1.1	0	0	0
	SMARCA2	2	8.8	6.7	5.3	0	2	0.9	0.6	0	2.7	1	0	0	1.7	0	0	0
	ARID1B	1.9	4.4	7.9	6.4	0	0	0.9	2.4	0	1.4	0	2.1	0	2.3	0	0	0
	SMARCC2	1.5	5.3	7.9	2.1	0	0.7	0	1.2	2.6	1.4	3	0	0	0	1.7	0	0
	ACTB	1.3	4.4	4.5	4.3	0	0	0	0	5.1	2.7	0	2.1	0.5	0	0	0	0
TP53	*TP53*	15.9	34.5	59.6	56.7	32	8	2.7	1.2	0	1.4	4	2.1	15.9	11.3	4.3	1.4	0
	ATM	5.5	13.3	12.4	15	4	5.3	3.6	3	0	2.7	5.1	0	1.5	6.2	4.3	0	1.3
	RB1	4.4	10.6	13.5	27.8	2	2	0	0	0	0	0	0	0.5	0.6	0.9	0	0
	CDKN1A	2.3	15	5.6	9.1	4	0	0	0.6	0	0	0	0	0	1.1	0	0	0
	FBXW7	1.8	5.3	6.7	10.7	0	0	0	0.6	0	0	0	2.1	0	0	0	0	0
	CDKN2A	1.5	6.2	2.2	9.1	0	0	0	0	0	0	1	0	0	0	0.9	0	0
RTK	*FGFR3*	3.4	37.2	3.4	5.9	0	0.7	0.9	0	2.6	0	4	0	0	0.6	0	0	0
	ERBB2	3.3	11.5	6.7	16	0	0.7	0.9	1.2	0	1.4	1	4.3	0.5	1.1	0	1.4	0
	ERBB3	2.7	14.2	9	10.2	2	0	0.9	1.2	0	0	1	0	0.5	0.6	1.7	0	0
	MET	2.5	3.5	3.4	5.3	0	0.7	0.9	0.6	5.1	2.7	6.1	21.3	0	1.7	0.9	0	0
	KRAS	1.9	5.3	4.5	2.1	0	1.3	0	0	0	0	0	10.6	0	1.1	0	17.4	0
	KIT	1.8	0.9	4.5	3.7	0	0	0	0	0	0	0	0	0	1.1	0	30.4	0
	BRAF	1.3	4.4	4.5	2.1	0	0	0.9	0	0	0	2	4.3	0.5	2.8	0.9	0	0
	HRAS	1.2	5.3	7.9	2.7	0	0	0.9	0	0	0	0	0	1	0.6	0.9	0	0
MTOR	*PIK3CA*	6.2	23.9	19.1	23	0	4	1.8	2.4	0	0	2	0	3.1	2.8	0	4.3	0
	PTEN	3.7	2.7	4.5	4.3	12	9.3	0	4.2	5.1	6.8	2	0	5.1	3.4	1.7	0	0
	MTOR	2.9	3.5	2.2	3.7	0	8	2.7	10.3	2.6	0	1	4.3	0.5	0.6	0	0	0
	TSC1	2.3	8.8	9	8	2	2	0	0.6	0	0	2	4.3	0.5	0	0	0	0
	TSC2	1.4	2.7	3.4	1.6	4	0.7	2.7	0	5.1	1.4	2	0	0.5	1.1	0.9	0	0
NRF2	*NFE2L2*	2.1	0.9	9	8	0	2.7	0	0.6	0	6.8	3	0	0	0.6	0	0	0
	CUL3	1.6	2.7	2.2	2.1	0	0	0.9	2.4	0	1.4	2	4.3	0.5	1.7	0.9	1.4	0
HIPPO	*NF2*	1.6	0.9	1.1	4.8	0	2	0	1.2	7.7	2.7	5.1	0	0.5	0.6	0	0	0
other BLCA	*STAG2*	3.9	23.9	9	10.7	0	4	0.9	0.6	0	1.4	7.1	0	0	0	0	0	1.3
	ELF3	2.9	15.9	16.9	9.1	0	0.7	0	0	0	4.1	0	0	0	0	0	0	0
	ERCC2	2.3	4.4	14.6	10.2	0	0	0	0	2.6	2.7	1	0	0.5	1.1	0	0	0
	BRWD1	2.1	4.4	6.7	5.3	0	2	1.8	1.2	2.6	1.4	3	2.1	1.5	1.1	0	0	0
	ZFP36L1	1.9	8	6.7	8	0	0.7	0	0.6	0	1.4	0	0	1	0.6	0	0	0
	ERBB4	1.8	4.4	0	3.7	2	4	1.8	2.4	0	2.7	1	0	1	1.1	0.9	0	0
	RHOB	1.5	6.2	10.1	4.3	0	0.7	0.9	0	0	0	1	0	0	0.6	0	0	0
	TXNIP	1.5	1.8	5.6	4.8	0	0.7	1.8	1.2	0	1.4	2	4.3	0.5	1.1	0	0	0
	RXRA	1.4	8.8	7.9	3.2	0	0	0	0	2.6	0	2	0	0	0.6	0	0	0
	KLF5	1.3	6.2	6.7	4.3	0	0	0	0	0	0	1	0	0	0.6	0.9	0	0
	FAM47C	1	2.7	4.5	1.6	2	0.7	0	0.6	0	1.4	1	2.1	0	2.3	0	0	0
other RCC	*VHL*	11.8	0.9	0	0.5	2	42.7	45.5	55.8	0	0	3	0	0.5	1.1	0	0	0
	BAP1	4	5.3	5.6	2.1	0	22.7	0.9	4.8	2.6	4.1	8.1	4.3	0	0	0	0	1.3
	STAG2	3.9	23.9	9	10.7	0	4	0.9	0.6	0	1.4	7.1	0	0	0	0	0	1.3
other PRAD	*BRCA2*	3.4	9.7	11.2	10.2	0	2	2.7	1.8	5.1	2.7	3	4.3	1.5	1.7	0.9	0	0
	SPOP	3.3	3.5	0	1.1	0	0.7	0	0	0	0	0	0	0.5	23.7	8.7	0	0
	CDK12	2.5	8	11.2	5.3	0	1.3	1.8	1.2	0	0	0	4.3	0.5	4	0	1.4	0
	FOXA1	2.3	4.4	3.4	2.1	0	0	0	0	0	0	1	0	0.5	13.6	1.7	1.4	1.3
	MED12	1.9	2.7	9	5.9	0	0.7	0	1.2	0	0	2	4.3	1	1.7	0.9	0	0
	CHD1	1.7	5.3	2.2	5.3	0	1.3	0	0	2.6	4.1	0	0	0	3.4	0.9	1.4	0
	CTNNB1	1.5	3.5	3.4	2.1	2	0	0	0.6	0	1.4	0	2.1	2.6	3.4	0	0	0
	ZMYM3	1.3	1.8	3.4	3.2	0	0	0	0.6	0	1.4	1	0	2.1	2.8	0.9	0	0

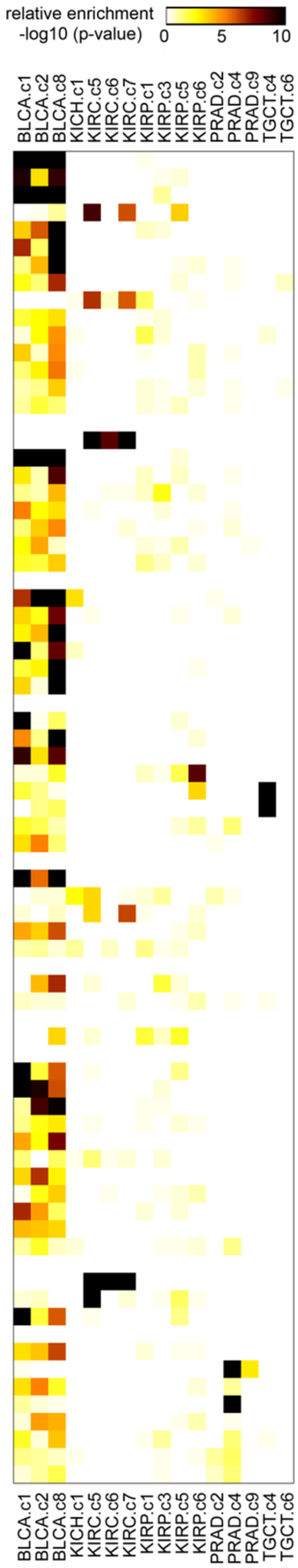

Supplementary Figure 4, related to Figure 4. Somatic mutations across pan-urologic genomic subtypes present within major cancer types. By TCGA project and genomic subtype (where projects were highly represented within a given subtype), percentages of somatic mutation for each indicated gene. Categories for selected genes are indicated off to the left. “Other BLCA,” significantly mutated genes from ref[1] not represented in the other categories; “other RCC,” significantly mutated genes from ref[2] not represented in the other categories; “other PRAD,” significantly mutated genes from ref[3] not represented in the other categories. For each TCGA project and genomic subtype category, the percentage reflects the number of mutation events found, versus the total number of cases for that category. Panel on the right represents significance of enrichment (one-sided Fisher’s exact test) of gene alteration events for each gene within any particular genomic subtype/cancer type versus the rest of the tumors.

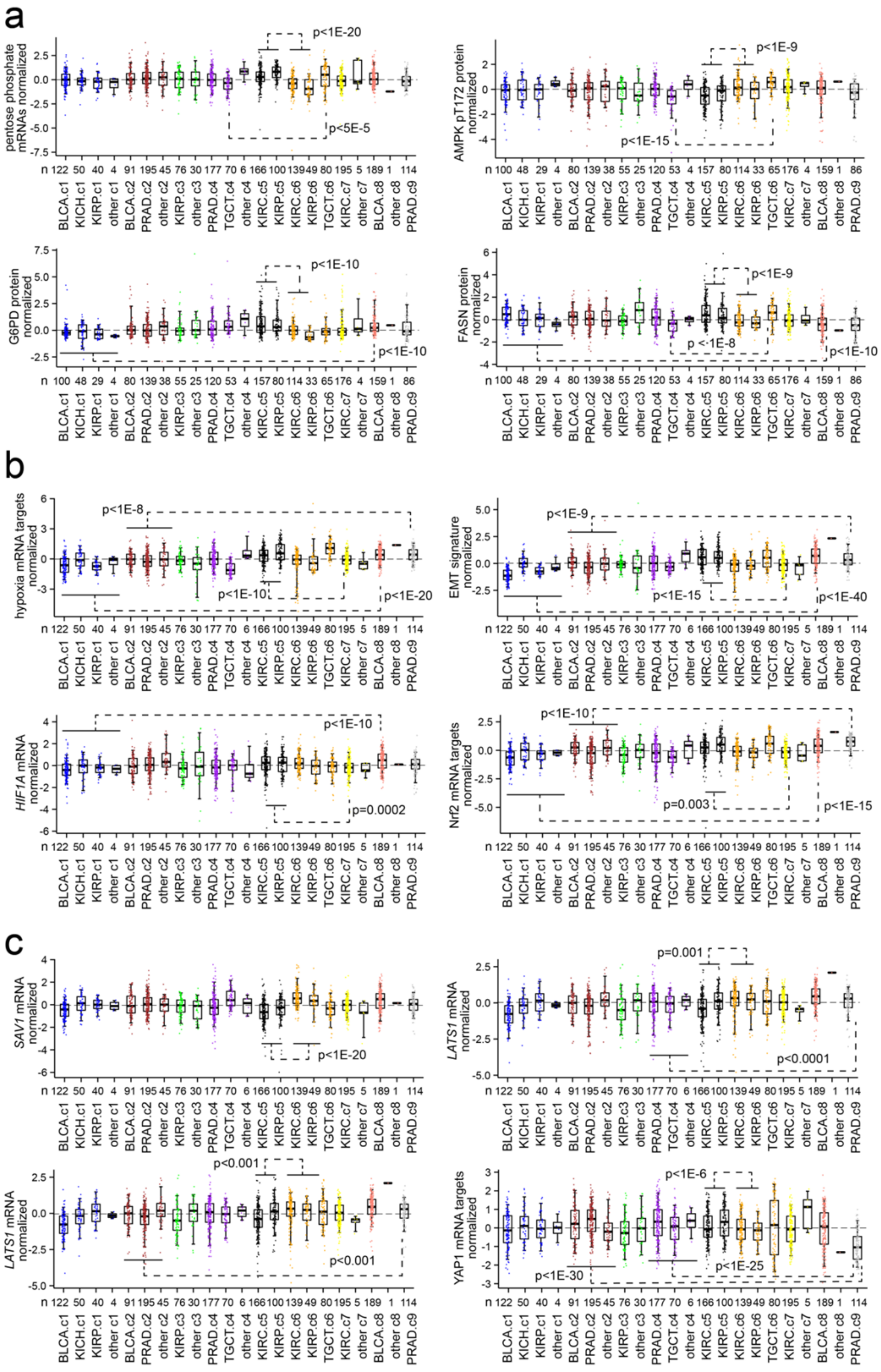
a
pentose phosphate mRNAs normalized
p<1E-20
p<5E-5
AMPK pT172 protein normalized
p<1E-9
p<1E-15
G6PD protein normalized
p<1E-10
p<1E-10
FASN protein normalized
p<1E-9
p <1E-8
p<1E-10
b
hypoxia mRNA targets normalized
p<1E-8
p<1E-10
p<1E-20
EMT signature normalized
p<1E-9
p<1E-15
p<1E-40
HIF1A mRNA normalized
p<1E-10
p=0.0002
Nrf2 mRNA targets normalized
p<1E-10
p=0.003
p<1E-15
c
SAV1 mRNA normalized
p<1E-20
LATS1 mRNA normalized
p=0.001
p<0.0001
LATS1 mRNA normalized
p<0.001
p<0.001
YAP1 mRNA targets normalized
p<1E-6
p<1E-30
p<1E-25
n
BLCA.c1
KICH.c1
KIRP.c1
other c1
BLCA.c2
PRAD.c2
other c2
KIRP.c3
other c3
PRAD.c4
TGCT.c4
other c4
KIRC.c5
KIRP.c5
KIRC.c6
KIRP.c6
TGCT.c6
KIRC.c7
other c7
BLCA.c8
other c8
PRAD.c9

Supplementary Figure 5, related to Figure 5. Differential levels of pathway features across pan-urologic genomic subtypes and cancer types. Features selected from main Figure 6 (values normalized within cancer type). **(a)** Pathway features associated with metabolic pathways. **(b)** Pathway features associated with hypoxia, EMT, MAPK, and NRF2/KEAP1. **(c)** Pathway features associated with HIPPO pathway. P-values by t-test, using log-transformed values centered within cancer type. Box plots represent 5%, 25%, 50%, 75%, and 95%.

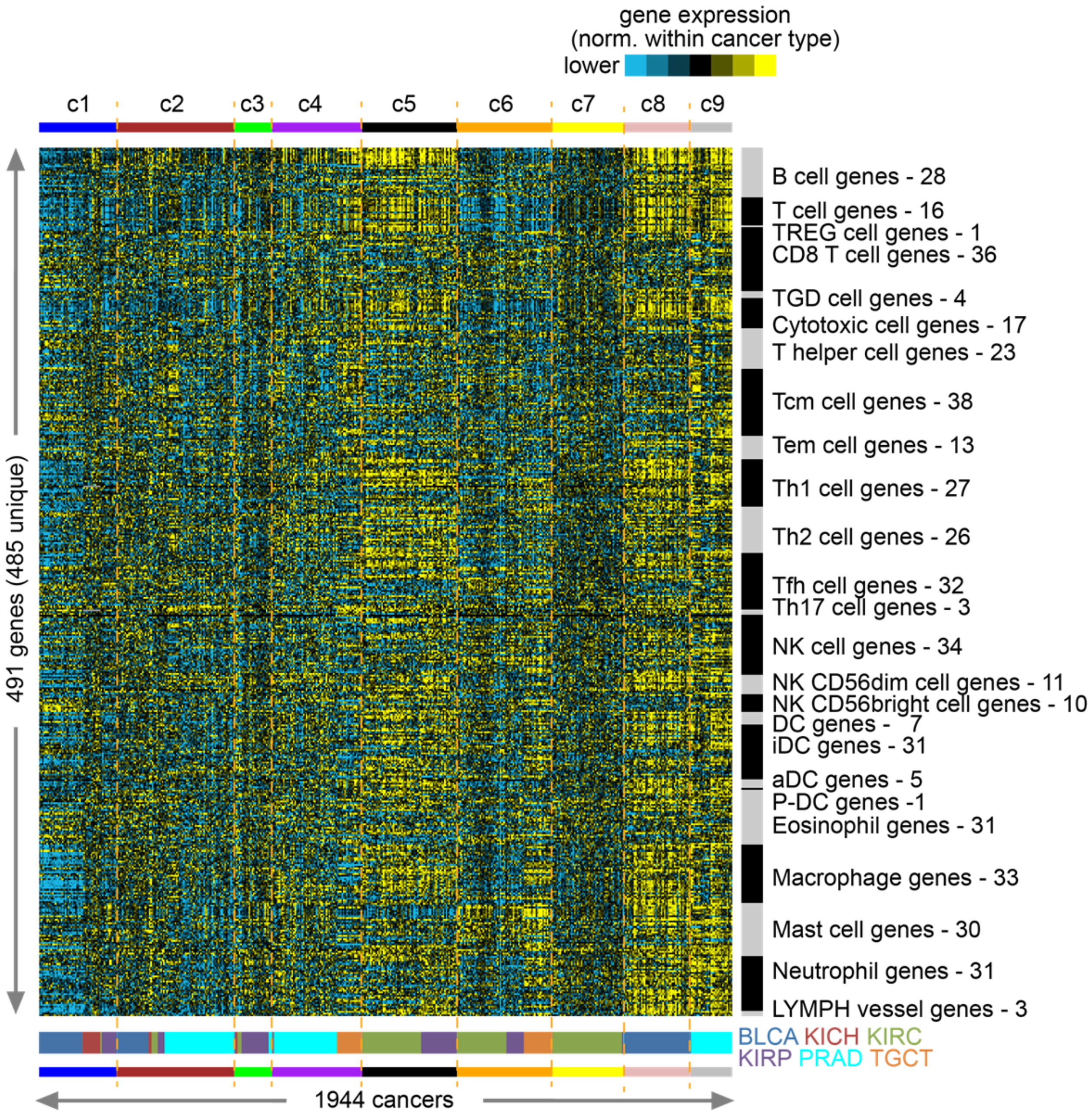

Supplementary Figure 6, related to Figure 6. Bindea immune-related expression signatures across pan-urologic genomic subtypes. Across TCGA urologic cancer subtypes (expression values normalized within cancer type), heat map of differential expression, for the individual genes in expression-based signatures of immune cell infiltrates (from Bindea *et al*.[4]). For each tumor profile, the normalized values within a given signature were averaged in order to derive a signature score (Figure 6b). TREG cells, regulatory T cells; TGD cells, T gamma delta cells; Tcm cells, T central memory cells; Tem cells, T effector memory cells; Tfh cells, T follicular helper cells; NK cells, natural killer cells; DC, dendritic cells; iDC, immature DCs; aDC, activated DCs; P-DC,

plasmacytoid DCs; APM1/APM2, antigen presentation on MHC class I/class II, respectively.

REFERENCES

1. Kim, J., *et al.* Invasive Bladder Cancer: Genomic Insights and Therapeutic Promise. *Clin Cancer Res* **21**, 4514-4524 (2015).
2. Chen, F., *et al.* Multilevel Genomics-Based Taxonomy of Renal Cell Carcinoma. *Cell Rep* **14**, 2476-2489 (2016).
3. Cancer_Genome_Atlas_Research_Network. The Molecular Taxonomy of Primary Prostate Cancer. *Cell* **163**, 1011-1025 (2015).
4. Bindea, G., *et al.* Spatiotemporal dynamics of intratumoral immune cells reveal the immune landscape in human cancer. *Immunity* **39**, 782-795 (2013).
